# *Drynaria fortunei* Promoted Angiogenesis Associated With Modified MMP-2/TIMP-2 Balance and Activation of VEGF Ligand/Receptors Expression

**DOI:** 10.3389/fphar.2018.00979

**Published:** 2018-09-21

**Authors:** Sheng-Teng Huang, Cheng-Chieh Chang, Jong-Hwei S. Pang, Hung-Sen Huang, Shen-Chieh Chou, Ming-Ching Kao, Huey-Ling You

**Affiliations:** ^1^Department of Chinese Medicine, China Medical University Hospital, Taichung, Taiwan; ^2^School of Chinese Medicine, China Medical University, Taichung, Taiwan; ^3^Research Center for Traditional Chinese Medicine, Department of Medical Research, China Medical University Hospital, Taichung, Taiwan; ^4^Chinese Medicine Research Center, China Medical University, Taichung, Taiwan; ^5^Research Center for Chinese Herbal Medicine, China Medical University, Taichung, Taiwan; ^6^Department of Chinese Medicine, Chang Gung Memorial Hospital–Kaohsiung Medical Center, Kaohsiung, Taiwan; ^7^Graduate Institute of Clinical Medical Sciences, Chang Gung University, Taoyuan, Taiwan; ^8^Department of Physical Medicine and Rehabilitation, Chang Gung Memorial Hospital, Taoyuan, Taiwan; ^9^Department of Pharmacy, School of Pharmacy, China Medical University, Taichung, Taiwan; ^10^Department of Biological Science and Technology, College of Biopharmaceutical and Food Science, China Medical University, Taichung, Taiwan; ^11^Department of Laboratory Medicine, Chang Gung Memorial Hospital–Kaohsiung Medical Center and Chang Gung University College of Medicine, Kaohsiung, Taiwan

**Keywords:** *Drynaria fortunei*, angiogenesis, matrix metalloproteinases (MMPs), VEGFs, VEGFRs

## Abstract

**Background and Purpose:** *Drynaria fortunei* J. Sm (*D. fortunei*), known as Gu-Sui-Bu, is used in traditional Chinese medicine to treat common injuries, including bone fractures and bruising. The specific functional mechanisms of the angiogenic and endothelial cell migration properties of *D. fortunei* are currently unclear. Thus, the purpose of this study is to validate the potential angiogenic and cellular migration properties and related mechanisms by *D. fortunei* both *in vivo* and *in vitro*.

**Experimental Approach:** The present study investigates, both *in vivo* and *in vitro*, the wound healing effects of *D. fortunei* as associated with angiogenesis, specifically by the modulation of matrix metalloproteinases (MMPs) and upregulation of vascular endothelial growth factor (VEGF) ligand/receptors. In order to determine the potential angiogenic effects of *D. fortunei*, *in vivo* neovascularization of chick chorioallantoic membranes (CAMs) assay, and directed *in vivo* angiogenesis assay (DIVVA) were performed, while *in vitro* scratch wound healing, migration, and matrix-induced tube formation assays were performed by using human umbilical vascular endothelial cells (HUVECs). Furthermore, we used qPCR to analyze the gene expressions and Western blot to observe protein expressions of MMP-2, MMP-14, TIMP-2, RECK, and VEGF/VEGFRs.

**Results:** This study identified five major compounds from the water extract of *D. fortunei*: protocatechuic acid, caffeic acid 4-*O*-β-D-glucopyranoside, 5,7-dihydroxychromone-7-*O*-rutinoside, neoeriocitrin, and naringin. *D. fortunei* was confirmed to activate *in vivo* angiogenesis by CAM and DIVVA assays. *D. fortunei* further exhibited *in vitro* angiogenic effects associated with cell migration, as demonstrated by the tube formation assay, transwell migration assay, and scratch wound healing assay. The extracellular MMP-2 activity was found to be dose-dependently augmented both *in vitro* and *in vivo* by *D. fortunei*. The mRNA and protein expressions of MMP-2, and MMP-14 were increased; while the tissue inhibitor metalloproteinase-2 (TIMP-2), and reversion-inducing cysteine-rich protein with kazal motifs (RECK) were both decreased. Furthermore, *D. fortunei* activated the gene and protein expressions of VEGF-A, -B, and VEGFR-2, -3.

**Conclusion:** *D. fortunei* increased MMP-2 activity, thereby stimulating angiogenesis and cell migration, both *in vivo* and *in vitro*, as a result of MMP-2 and TIMP-2 balance modulation and the activation of VEGF/VEGFRs expression.

## Introduction

Gu-Sui-Bu (*Drynaria fortunei*) is a species of fern native to Eastern Asia often used to treat traumatism in TCM. According to the *Compendium of Materia Medica* (Ben Cao Gang Mu), the most complete and comprehensive medical text concerning TCM, Gu-Sui-Bu is traditionally used to treat bone fractures, skin abrasions, cuts, bruises, and toothache. It is important to note that there are various species of Gu-Sui-Bu that have been documented and used in the treatment of various ailments. The germplasm resources of Gu-Sui-Bu belong to the *Polypodiaceae* and *Davalliaceae* plant families; of these, *Drynaria fortunei* J. Sm (*Polypodiaceae*) (*D. fortunei*) is the most frequently used species of Gu-Sui-Bu, commonly prescribed in TCM for the treatment of the above-mentioned conditions (Wong and Rabie, 2006; Yang et al., 2014). The dry stem and root of *D. fortunei* are applied to the clinical medical use. It is a popular TCM herb used by tea drinking or dressing for topical use. Several studies have demonstrated the effects of *D. fortunei* on bone-related diseases. One study in particular has demonstrated that *D. fortunei* improved the bone mass of ovariectomized rats (Lee et al., 2014); further, the flavonoid fractions extracted from *D. fortunei* exerted estrogen-like protective effects in bone tissue (Wong et al., 2013). Additionally, it has been shown to stimulate stem cell proliferation and osteogenic differentiation (Huang et al., 2012). Furthermore, studies have also illustrated that *D. fortunei* enhances osteoblast activity and inhibits osteoclast functions (Sun et al., 2004; Li et al., 2006; Wang et al., 2011; Zhu et al., 2012). Despite various studies demonstrating the effects of *D. fortunei*, the specific mechanisms associated with the angiogenic properties are as yet unclear.

Formation of new blood vessels, through either angiogenesis or vasculogenesis, is a fundamental biological function essential to the wound healing process, dependent on sufficient oxygen and nutrition supplies (Tonnesen et al., 2000). The wound healing process consists of replacing devitalized tissue and recovering cellular structures. As such, the adult human wound healing process can be divided into three distinct phases: inflammation, proliferation, and remodeling (Maxson et al., 2012). During wound healing and/or angiogenesis, inflammation responses stimulate the release of growth factors, such as VEGFs and MMPs, which attract stem cells and promote angiogenesis.

Matrix metalloproteinases are a family of enzymes that are subdivided into six groups: collagenases, gelatinases, membrane type, stromelysins, matrilysins, and others (Verma and Hansch, 2007); they can further be expressed in macrophages, vascular smooth muscle cells, lymphocytes, and endothelial cells (Newby, 2006). The MMPs and TIMPs play a significant role in regulating angiogenesis. A variety of cytokines and growth factors regulate the expression of MMPs and TIMPs (Clark et al., 2008). The MMPs are also implicated in the wound healing process by degrading the ECM (Newby, 2006). The major MMPs involved in the process of angiogenesis are MMP-2 and MMP-14 (Stetler-Stevenson, 1999; Verma and Hansch, 2007; Hung et al., 2010). MMP-2 activity can be regulated by other MMPs (e.g., MMP-2, MMP-3, MMP-9, MMP-14) and MMP inhibitors (e.g., TIMP-2 and RECK) (Stetler-Stevenson, 1999; Oh et al., 2001; Bjorklund and Koivunen, 2005). Under normal conditions, pro-MMP-2, TIMP-1, and TIMP-2 are subtly balanced; however, this balance may be affected during the process of angiogenesis. When this occurs, the MMP activity can be dramatically enhanced.

During the wound healing process, a dynamic interaction occurs among the ECM environment, regulated by angiogenic cytokines such as fibroblast growth factors (FGFs) and VEGF. The interaction between VEGF and VEGFR is also a major contributor to angiogenesis (Chodorowska et al., 2004), which could act to increase the number of capillaries in a given network. In mammals, the VEGF family is comprised of five members: VEGF-A, PGF, VEGF-B, VEGF-C, and VEGF-D (Hoeben et al., 2004). Of these, VEGF-A, has been shown to stimulate endothelial cell mitogenesis and cell migration, and is the most important VEGF for angiogenesis (Egeblad and Werb, 2002; Holmes and Zachary, 2005). VEGF-A is the key inducer of developmental angiogenesis via activation of VEGF receptors 1 and 2 (VEGFR-1, -2), it is also a vasodilator, increasing microvascular permeability (Davis et al., 2010). VEGFR-2 appears to mediate almost all of the known cellular responses to VEGF (Holmes et al., 2007); while the function of VEGFR-1 is less well-defined, it is thought to modulate VEGFR-2 signaling (Zygmunt et al., 2011; Eklund et al., 2013). In contrast to VEGF-A, VEGF-B plays a less significant role in the vascular system. It is thought to play a role not only in the maintenance of newly formed blood vessels during pathological conditions, but also in the protection of neurons (Poesen et al., 2008; Zhang et al., 2009). VEGF-C and VEGF-D are ligands for VEGFR-3, which mediates lymphangiogenesis; it has been reported that VEGF-C can stimulate lymphangiogenesis via VEGFR-3, and angiogenesis via VEGFR-2 (Paavonen et al., 2000).

The specific angiogenic mechanisms responsible for the cell migration properties of *D. fortunei* remain unclear. The present study investigates the potential angiogenic and cellular migration properties of *D. fortunei* by utilizing both *in vivo* and *in vitro* experimental models, and further explores the molecular mechanisms related to the expressions of MMP-2, TIMP-2, RECK, and VEGF/VEGFRs.

## Materials and Methods

### Preparation of Water Extract of *D. fortunei*

The *D. fortunei* used in this study was identified based on the definition described in Flora of Taiwan. A sample of *D. fortunei* is deposited in Set D, TN: No 68470 in the herbarium of National Taiwan University. The extract was prepared according to Taiwanese GMP methodologies and guidelines. Briefly, the whole plant was minced and extracted with boiling water 1:20 (w/v) for 4 h; following which, the extract was removed. Then water 1:20 (w/v) was again added and boiled for 4 h. The resulting crude extract was filtered and lyophilized down to dry powder. The extract used in the experiments was prepared by dissolving the powder in sterile water at the desired concentration (100 mg/mL). The extraction rate was 27.4%, i.e., 1 g of lyophilized powder is equal to 3.65 g original herb.

### Chromatographic Procedure

The HPLC system consisted of a Shimadzu LC-20AT and SPD-20A UV-Vis detector. Chromatographic separation was performed on Phenomenex^®^ Luna (2) C-18 (4.6 mm × 250 mm ID, 5 μm). The mobile phase consisted of a mixture of water + 0.5% acetic acid (A) and methanol (B) using a gradient elution programmed as follows: 0 min: 10% B, 10 min: 39% B, 20 min: 47% B, 25 min: 47%B, 30 min: 10%B, and 40 min: 10%B, with a flow rate of 1.0 mL/min. Detection wavelength was set at 270 nm and injection volume was 20 μL. Quantitative analysis was completed via external calibration method by the establishment of a calibration curve with peak areas under curves. The amount of protocatechuic acid, neoeriocitrin, and naringin were determined over extract with *R*^2^ > 0.99 as 0.07%, 1.62%, and 1.48% (w/w), respectively. Additionally, we purchased caffeic acid as a substituent for caffeic acid β-D-glucopyranoside because the extinction coefficient is quite close as both share the same chromophore. We prepared 0.0038, 0.0069, 0.0139, 0.028, and 0.056 μmole as establishment of calibration curve of caffeic acid with 1,000 μg injection of *D. fortunei* extract. The equivalent of caffeic acid standard for caffeic acid β-D-glucopyranoside is 2.46% (w/w). The LC-MS was performed by a UHPLC system (Ultimate 3000; Dionex, Germany) equipped with a C18 reversed-phase column (Waters T3 Column, 100 Å, 3.0 μm, 2.1 mm × 150 mm) coupled with a hybrid Q-TOF mass spectrometer (maXis impact, Bruker Daltonics, Bremen, Germany) with an orthogonal ESI source. The initial flow rate was 0.2 L/min of 95% solvent A (0.1% formic acid) and 5% solvent B (100% methanol). A volume of 5 μL of sample was injected. After injection, solvent B was increased to 50% at 12 min, linearly increased to 95% at 14 min, held at 95% for another 1 min, then decreased to 5% at 16 min, and held at 5% for 4 min. The mass spectrometer was operated in negative ion mode using the m/z range 50∼1000 at 2 Hz. The relative capillary voltage of the ion source was -2,200 V for negative mode, and the endplate offset was 500 V. The nebulizer gas flow was 1 bar and drying gas flow was 8 L/min. The drying temperature was set at 200°C.

### Cell Culture

Human umbilical vascular endothelial cells were obtained from BCRC (Bioresource Collection and Research Center, Taiwan, ROC. Catalog Number: H-UV001) and grown in EGM provided by Clonetics (Walkersville, MD, United States). Cells were maintained in a humidified atmosphere with 5% CO_2_/95% air at 37°C and were passaged three to five times prior to use in experiments. To examine the effect of *D. fortunei* on cell function, cells at 80–90% confluency were treated with 0.5–5 mg/ml *D. fortunei* for 24 h before experiments. Protocatechuic acid (PA) at 1–25 μM was tested for cytotoxicity, wound healing, migration and capillary tube formation.

### Cell Viability Assay

Cells with or without *D. fortunei* (0.5, 1, 5 mg/ml) and PA (1, 5, 10, 25 μM) treatment for 24 h were washed once with PBS, followed by the addition of 1 ml DMEM containing 0.05 mg/ml 3-(4-,5-dimethylthiazol-2-yl)-2, 5-diphenyltetrazolium bromide (MTT). After incubation at 37°C for 1 h, the media was removed and the formazan crystals in the cells were solubilized in 1 ml DMSO for OD reading at 570 nm using a spectrophotometer.

### Cytotoxicity Assay

Lactate dehydrogenase activity was measured using the CytoTox 96^®^ Non-Radioactive Cytotoxicity Assay (Promega, Mannheim, Germany). Disruption of plasma membrane integrity leads to a release of LDH into the supernatant and results in the conversion of a tetrazolium salt into a red formazan product. HUVECs were treated with 0.5–5 mg/ml *D. fortunei* for 24 h and 50 μl of the supernatant was collected, and the LDH activity was measured and read at 490 nm using a spectrophotometer.

### *In vivo* Cultrex Assay

A DIVAA (Trevigen, Gaithersburg, MD, United States) was performed as described previously, with slight modifications (Basile et al., 2007). Briefly, angioreactors were filled with 18 μl of Cultrex-reconstituted basement membrane substrate (Trevigen) containing 37.5 ng of VEGF and 12.5 ng of basic fibroblast growth factor as a positive control. There were three mice included *in vivo* of DIVVA for each group with two independent experiments. Each mouse would be implanted with two angioreactors for angiogenic observation. These angioreactors were implanted subcutaneously into the dorsal area of 6-week-old NOD SCID mice (BioLASCO, Taiwan). Two days after implantation, the mice began to feed with 250 mg/kg or 500 mg/kg of *D. fortunei* for 15 days. The doses of 250 and 500 mg/kg to the treatment of mice based on the previous studies (Wong et al., 2013; Lee et al., 2014). As for the negative and positive control groups, the mice were only fed with water instead of *D. fortunei.* Then, the mice of these four groups were sacrificed and the angioreactors removed, photographed, and processed with fluorescein isothiocyanate-labeled Griffonia lectin, an endothelial cell-selective reagent, to quantify invasion of endothelial cells into the angioreactors. Fluorescence was determined in a plate reader as mean relative fluorescence units for duplicate assays. All experiments were approved by Institutional Animal Care and Use Committee (Approval No. 2016-333-1) at China Medical University Hospital (Taichung, Taiwan).

### CAM Angiogenesis Assay

Angiogenesis assays were performed on the CAM of 10-day-old chick embryos with minor modifications (Brooks et al., 1994). First, a small hole was made through the shell at the air sac end of the chick egg using a small craft drill. A second hole was drilled on the broad side of the egg directly over the embryonic blood vessels. Negative pressure was applied to the original hole resulting in the pulling of CAM away from the shell membrane and formation of a false air sac. The shells were covered with adhesive tape and incubated in a 37°C incubator. After 48 h, the CAM tissues were harvested and examined for angiogenesis under a stereomicroscope (Zeiss, Germany). The angiogenic index was defined as the mean number of visible blood vessel branch points within the defined area of the membrane. Photographs were taken at 16× magnification (Tsai et al., 2011). For each CAM angiogenesis assay, we conducted a triplicate (three eggs) for each dose (0.5–5 mg/ml), with each independent experiment performed three times.

### *In vitro* Capillary Tube Formation Assay

The ECM gel-induced capillary tube formation assay was used as an *in vitro* measurement of angiogenesis. Briefly, a 24-well culture plate was coated with 75 μl/well Matrigel ECM gel (11.34 mg/ml) prepared from Engelbreth Holm-Swarm mouse sarcoma (Sigma) and allowed to form a gel layer for 30 min at 37°C. After gel formation, 7 × 10^4^ HUVECs in 0.5 ml of growth medium were seeded to each well with or without treatment of *D. fortunei* and PA. The plate was incubated at 37°C in a humidified atmosphere with 5% CO_2_/95% air for 4 h and the formation of capillary tubes were photographed with the use of an inverted microscope. The formation of capillary tubes was quantified by counting the number of tubes with the Wimtube program (Wimasis Image).

### Transwell Filter Migration Assay

Transwell filters (Costar, Cambridge, MA, United States) with 8.0 μm pores were used for migration assay. HUVECs cultured with or without treatment of *D. fortunei* and PA for 24 h were seeded at a density of 1.2 × 10^5^ cells per filter. To initiate the migration assay, cells in 250 μl M199 without FBS were added to the inner chamber, while the lower chamber was filled with 600 μl M199 and 10% FBS as an inducer of cell migration. Cells were allowed to migrate in an atmosphere of 95% air/5% CO_2_ at 37°C for 2 h. Cells on the filter were first stained with Liu’s stain and cells that remained on the upper surface of the filter were removed using a cotton swab. The cells that migrated onto the lower surface of the filter were examined by microscope after mounting on a slide. A total of six random HPFs (100X) per filter were photographed and the numbers of cells were counted by using ImageJ software.

### *In vitro* Scratch Wound Assay

Cells were counted and plated in equal numbers in 6-well tissue culture plates to achieve 90% confluence. Thereafter, a vertical wound was created using a 1 mL pipette tip. The cells were incubated in EGM-2 medium with or without treatment of *D. fortunei* and PA. The area of the cell-free wound was recorded with microscopy after 24 h incubation. Measurement of scratch wound was assessed by using ImageJ software.

### Zymography Analysis

Chick chorioallantoic membrane harvested from the egg was homogenized in 1x PBS. The supernatant was collected after brief centrifugation and used for the analysis of MMP activity. Conditioned media collected from cultured human endothelial cells with 24 h treatment with *D. fortunei* were processed for analysis of MMP activity. Samples with 10 μg of total proteins were loaded onto 10% sodium dodecyl sulfate polyacrylamide gel electrophoresis (SDS-PAGE) gels in which 1% gelatin (Amersham Life Science, Cleveland, OH, United States) was incorporated. After migration, the gels were incubated with 2.5% Triton-X 100 twice for 30 min at room temperature, washed for 5 min in TNCA (50 mM Tris pH 7.5, 200 mM NaCl, 5 mM CaCl_2_) and further incubated for 16 h in TNCA in a shaking bath at 37°C. The gels were stained for 1 h in coomassie blue (0.1% coomassie brilliant blue R-250, 50% methanol, 10% acetic acid) and destained in 5% methanol/9% acetic acid until the proper contrast was achieved. White bands on blue backgrounds indicated zones of digestion corresponding to the presence of different MMPs. Measurement of the bands was assessed by using ImageJ software.

### RNA Isolation and qRT-PCR

Total cellular RNA was isolated by lysis of cells in a guanidinium isothiocyanate buffer, followed by a single step phenol–chloroform–isoamyl alcohol extraction procedure, modified from that previously described (Chomczynski and Sacchi, 1987). Briefly, cells treated or untreated with *D. fortunei* were harvested and lysed in 4M guanidinium isothiocyanate, 25 mM sodium citrate (pH 7.0), 0.5% sodium sarcosine, and 0.1M β-mercaptoethanol. Sequentially, 1/10 volume of 2M sodium acetate (pH 4.0), one volume of phenol and 1/5 volume of chloroform–isoamyl alcohol (49:1, v:v) were added to the homogenate. After vigorous vortexing for 30 s, the solution was centrifuged at 10,000 × *g* for 15 min at 4°C. After removal of the aqueous phase, RNA was precipitated by the addition of 0.5 mL isopropanol. For qRT-PCR analysis, reverse transcription was performed using 1 μg of total RNA and oligo (dT) primers in a 20 μL reaction, according to the manufacturer’s protocol (PE Applied Biosystems, Foster City, CA, United States). Real-time PCR was performed using the Mx3005 qPCR system (Strategene, La Jolla, CA, United States) with SYBR green (Applied Biosystems) as a dsDNA-specific binding dye. The PCR was cycled 40 times after initial denaturation (95°C, 2 min) with the following parameters: denaturation, 95°C, 15 s; and annealing and extension, 60°C, 1 min. The threshold cycle (C_t_) (GAPDH 18–25 cycles, and other genes 25–30 cycles) was recorded for each sample to reflect the mRNA expression level. Sequences for the specific primers used in the PCR are shown in **Supplementary Table S1**.

### Western Blotting

Protein concentrations were determined by the Bradford method (Bio-Rad, Hercules, CA, United States). Samples with 25 μg of total proteins were subjected to 10% SDS-PAGE and transferred onto a polyvinylidene difluoride (PVDF) (Milli-pore, Bedford, MA, United States) membrane. The membrane was incubated at room temperature in blocking solution [1% BSA (bovine serum albumin), 1% goat serum in PBS] for 1 h, followed by 2 h of incubation in blocking solution containing an appropriate dilution (1:1000) of primary antibody, e.g., anti-MMP-2, anti-MMP-14, anti-TIMP-2, anti-RECK, anti-VEGFs, and anti-VEGFRs (NeoMarkers, Fremont, CA, United States). After washing, the membrane was incubated in PBS containing goat anti-mouse IgG (1:5000) conjugated with horseradish peroxidase (Sigma, St. Louis, MO, United States) for 1 h. The membrane was washed and the positive signals were developed with chemiluminescence reagent (Amersham Pharmacia Biotech, Little Chal-font Buckinghamshire, England). Measurement of the bands was assessed by using ImageJ software.

### Statistical Analysis

All statistical analyses were performed using SigmaStat statistical software (version 2.0, Jandel Scientific, SanRafael, CA, United States). Results were represented as means ± standard deviation (SD). ANOVA was carried out when multiple comparisons were evaluated. Values were considered to be significant at *p* less than 0.05. All experiments were repeated at least three times independently.

## Results

### The Fingerprint of *D. fortunei*

**FIGURE 1:**
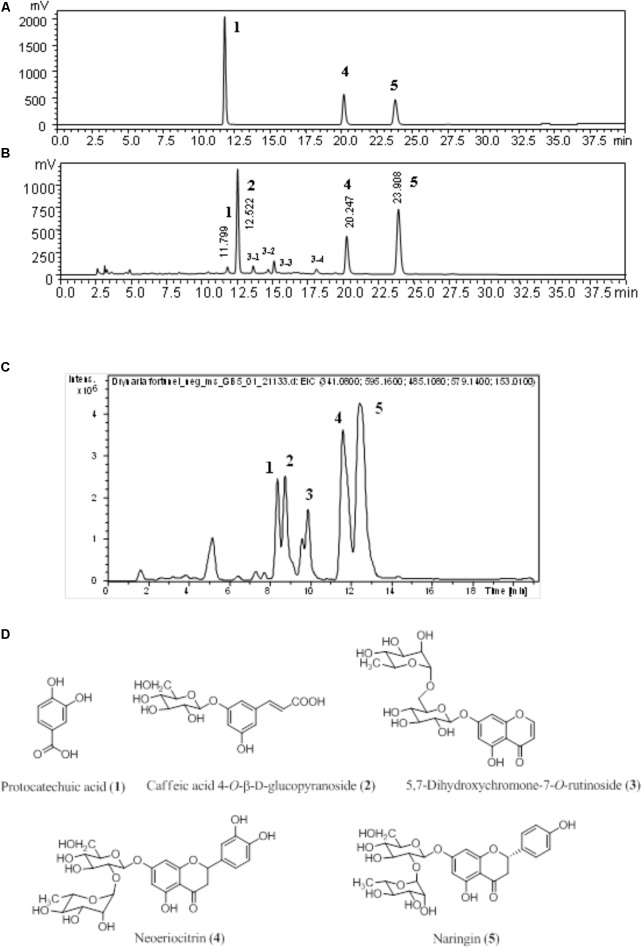
The chromatogram of *D. fortunei* aqueous extract. The authentic standards **(A)** and *D. fortunei* aqueous extract **(B)** of HPLC and EIC **(C)** with identification of extract included protocatechuic acid (**1**), caffeic acid 4-*O*-β-D-glucopyranoside (**2**), 5,7-dihydroxychromone-7-*O*-rutinoside (3), neoeriocitrin (**4**) and naringin (**5**). The structures of **1**-**5** were shown in **(D)**. The respective gradient program and column of HPLC **(A,B)** and UPLC **(C)**, see the section “Chromatographic Procedure.”

Fingerprinting is routine, crucial, and standard procedure of maintaining quality control for herbal medicine. The chromatogram detected at 270 nm of authentic standards, *D. fortunei* aqueous extract is shown in **Figures 1A,B**, respectively. Identification of the presence of protocatechuic acid, neoeriocitrin and naringin were confirmed by spiking with standards and comparing with literature (Liu et al., 2012). The extracted-ion chromatogram (EIC), shown in **Figure 1C**, for protocatechuic acid (**1**), caffeic acid 4-*O*-β-D-glucopyranoside (**2**), 5, 7-dihydroxychromone-7-*O*-rutinoside (**3**), neoeriocitrin (**4**), and naringin (**5**), were detected as negative ion mode with *m/z* at 153.01, 341.07, 485.10, 595.14, and 579.14, respectively. The UV-Vis spectra for **3**-**1**, **3**-**2**, **3**-**3**, and **3**-**4** were demonstrated in **Supplementary Figure S3**. Compound **3** is determined to be **3**-**3** in **Figure 1B** based on UV maximum absorption at 226, 249, 256, and 287 nm and fragment ion at m/z 177 which is characteristic ion for 7-dihydroxychromone. Additionally, the MS, MS/MS for **1**-**5** and base peak chromatography (BPC) for water extract of *D. fortunei* were shown in **Supplementary Figure S4**. All structures are shown in **Figure 1D**.

### No Cytotoxic Effect of *D. fortunei* and PA on HUVECs

To rule out possible cytotoxic effects of *D. fortunei* on HUVECs, cells were treated with *D. fortunei* at concentrations up to 5 mg/ml for 24 h. Cell viability was determined by standard MTT assay, and cytotoxic effect was evaluated by the LDH assay. The MTT assay measures activity of mitochondria as a means of assessing numbers of living cells (Denizot and Lang, 1986). The HUVECs’ viability was not significantly affected after treatment with *D. fortunei* (**Supplementary Figure S1A**). Furthermore, the LDH release did not change under the experimental conditions (**Supplementary Figure S1B**). Therefore, the HUVECs did not reveal any detrimental effects after *D. fortunei* treatment. Additionally, cell morphology under microscopic examination did not show any signs of apoptosis or necrosis after 5 mg/ml *D. fortunei* treatment for 24 h (data not shown). Meanwhile, no significant cytotoxicity to HUVECs was detected with treatments of PA at various concentrations (**Supplementary Figure S1C**).

### *In vivo* Angiogenesis Assays by *D. fortunei*

**FIGURE 2:**
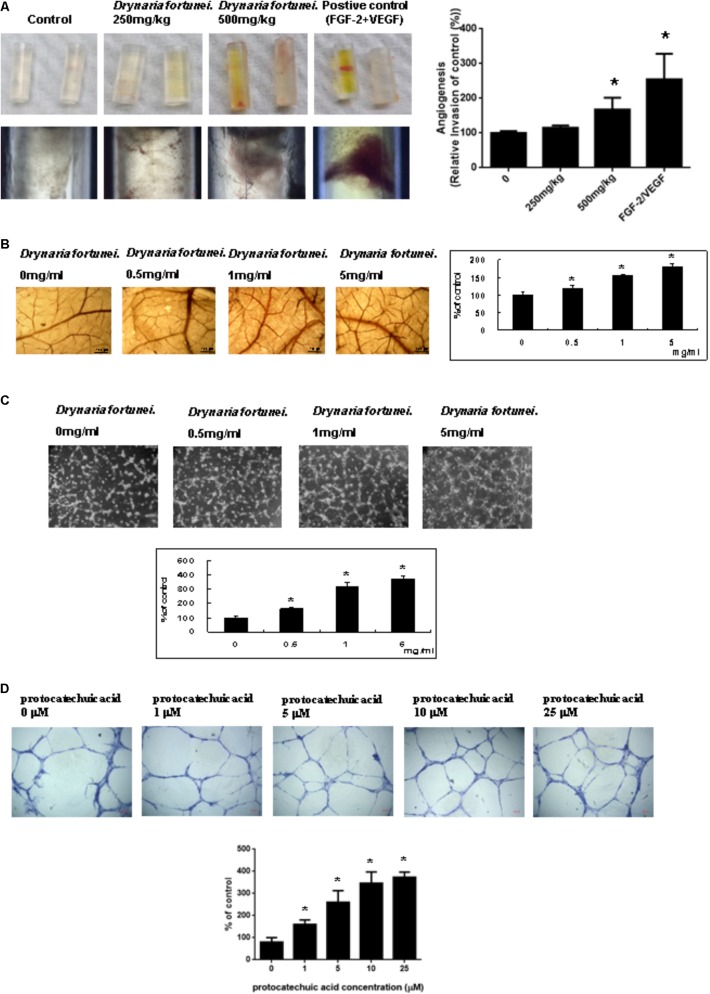
*D. fortunei* promoted angiogenesis both *in vivo* and *in vitro*. Angiogenesis assays were performed *in vivo* and *ex vivo* models. **(A)** Directed *in vivo* angiogenesis assay (DIVAA) showing silicone tubes was applied in this study. Angioreactors were implanted subcutaneously into the dorsal areas of nude mice for 2 days, followed by feeding with and without *D. fortunei* at 250 mg/kg and 500 mg/kg for 15 days to allow new vessels to infiltrate. Then, the mice were sacrificed and the angioreactors removed, photographed, and calculated. **(B)** The CAM of 10-day-old chick embryos was used as an *ex vivo* model. The vessel density of CAM was increased with a dose-dependent manner in line with the concentrations of *D. fortunei*. The CAM tissues were examined for angiogenesis under a stereomicroscope (magnification, ×16) as indicated concentrations after 48 h incubation. **(C)** *D. fortunei* increased ECM gel-induced capillary tube formation in a dose-dependent manner. HUVECs were seeded to the Matrigel ECM gel layer in 24-well culture plate with or without treatment of *D. fortunei* at various concentrations for 24 h. **(D)** PA as a positive control also enhanced ECM gel-induced capillary tube formation dose-dependently. HUVECs were seeded to the Matrigel ECM gel layer in 24-well culture plate with or without treatment of PA at various concentrations for 24 h. Data were mean ± SD calculated from three individual experiments. An asterisk demonstrated the significant difference (^∗^*P* < 0.05) compared with control.

The angiogenic effects of *D. fortunei* were further analyzed by DIVVA, as shown in **Figure 2A**. A significant increase in the invasion of vessels was noted in the angioreactors for the high-dose group (500 mg/kg) compared to the negative control and low-dose group (250 mg/kg), but less than the positive control. The estimated 60% vascularization of the tubes was augmented only in the high-dose group, but not in the low-dose or the negative control. Quantification of fluorescein isothiocyanate-lectin binding to the endothelial contents of the angioreactors indicated that *D. fortunei* may induce angiogenesis.

### The Chicken Embryo Chorioallantoic Membrane (CAM) Assay by *D. fortunei*

Chick chorioallantoic membrane assays have been widely used as an *ex vivo* model to study angiogenesis. After 24 h of incubation in the presence of different concentrations of *D. fortunei* (0, 0.5, 1, 5 mg/ml), the CAM tissues were examined for angiogenesis under a stereomicroscope (magnification, ×16), as shown in **Figure 2B**. Angiogenesis was scored under the stereomicroscope. The vessel density of the CAM was increased in a dose-dependent manner, up to 1.8 times, after treatment with 5 mg/ml solution of *D. fortunei* (**Figure 2B**).

### *D. fortunei* and PA Increased ECM Gel-Induced Capillary Tube Formation

Angiogenesis is assessed by measuring the ability of endothelial cells to form three-dimensional structures (tube formation). To this end, HUVECs were seeded to the matrigel ECM gel layer in 24-well culture plate with or without treatment of *D. fortunei* and PA at various concentrations for 24 h. 1 mg/ml *D. fortunei* water extract contained approximate 5 μM PA. Thus, we used PA as a positive control to compare with *D. fortunei*. The differentiation of HUVECs into an extensive and complete network of capillary-like structures was shown in the representative photomicrographs. The tube formation of HUVECs increased exponentially, up to nearly four times, with 5 mg/ml *D. fortunei* as well as 25 μM PA treatments (**Figures 2C,D**).

### The Scratch Wound Healing of HUVECs Was Enhanced by *D. fortunei* and PA

**FIGURE 3:**
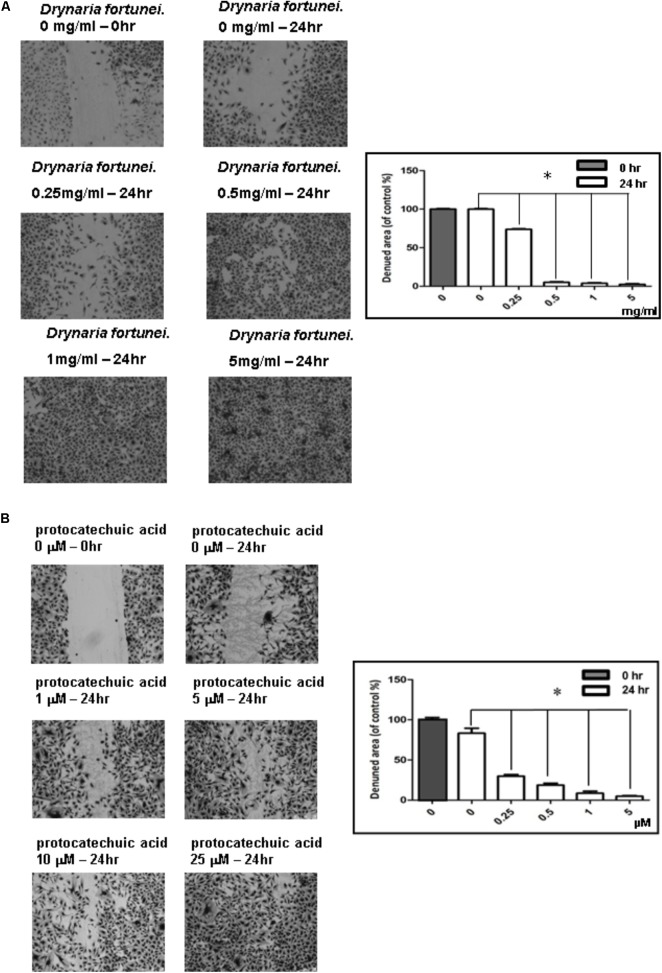
The scratch wound healing effect of HUVECs with *D. fortunei* and PA treatment. The cells were incubated in EGM-2 medium with different concentrations of *D. fortunei* and PA as indicated for 24 h. The vertical wound was created using a 1 mL pipette tip. **(A)** The wound healing effect was enhanced up to complete recovery with increasing concentrations of 5 mg/ml *D. fortunei*. **(B)** PA also promoted the wound healing dose-dependently. Data were mean ± SD calculated from three individual experiments. An asterisk demonstrated the significant difference (^∗^*P* < 0.05) compared with control.

The scratch wound healing assay is a simple method used to study cell migration and cell regrowth. The microscopic pictures revealed the presence of migration characteristics with different concentrations of *D. fortunei* and PA treatment. The HUVECs regrowth and migration from the wound edge was completely recovered after treatment with *D. fortunei* and PA for 24 h (**Figures 3A,B**).

### The Migration of HUVECs Was Enhanced With *D. fortunei* and PA

**FIGURE 4:**
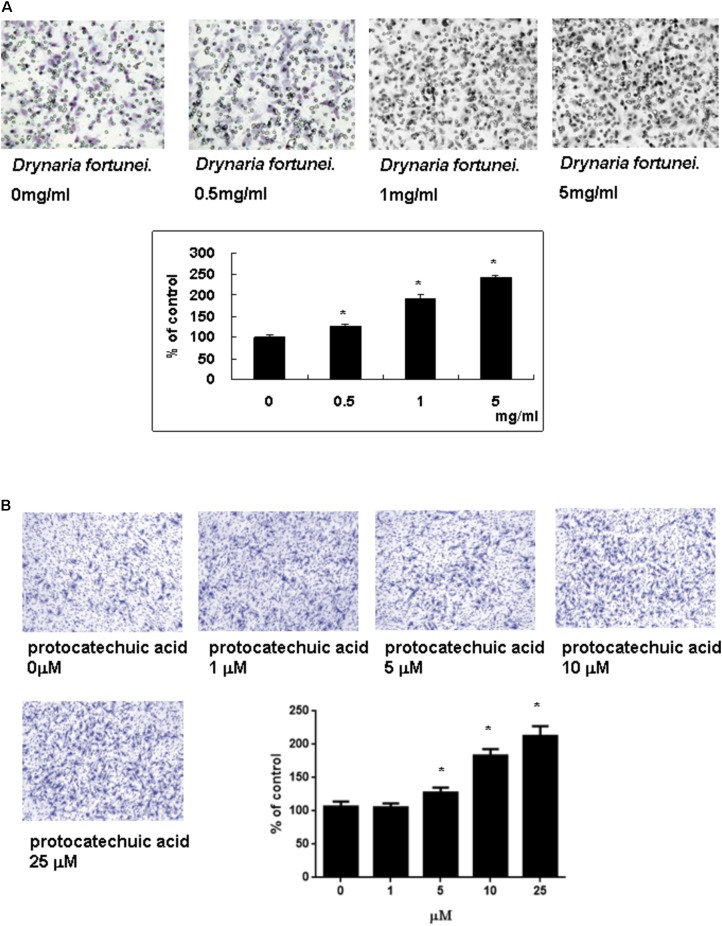
The *D. fortunei* and PA increased the migration ability of HUVECs. A Transwell chamber was used for the migration assay. HUVECs were treated with different concentrations of *D. fortunei* and PA for 24 h. The cells that migrated onto the lower surface of the filter were examined by microscope (magnification, ×100). Both of *D. fortunei* **(A)** and PA **(B)** stimulated the migration of HUVECs through the uncoated porous filter for 2 h with a dose-dependent manner paralleled with increasing concentrations. Data were mean ± SD calculated from three individual experiments. An asterisk demonstrated the significant difference (^∗^*P* < 0.05) compared with control.

The transwell migration assay was introduced in this study to show the migratory response of HUVECs. HUVECs were treated with different concentrations of *D. fortunei* and PA for 24 h. The cells which migrated onto the lower surface of the filter were examined by microscope (magnification, ×100). The migration of HUVECs was increased in a dose-dependent manner by both *D. fortunei* and PA. The migration degree was up to 2.5 times higher with the treatment of 5 mg/ml *D. fortunei* compared to the control without treatment (**Figure 4A**); whereas PA enhanced the migration activity up to two times, compared to vehicle controls (**Figure 4B**).

### The Gelatin Zymography of MMP-2 Activity of CAMs and HUVECs

**FIGURE 5:**
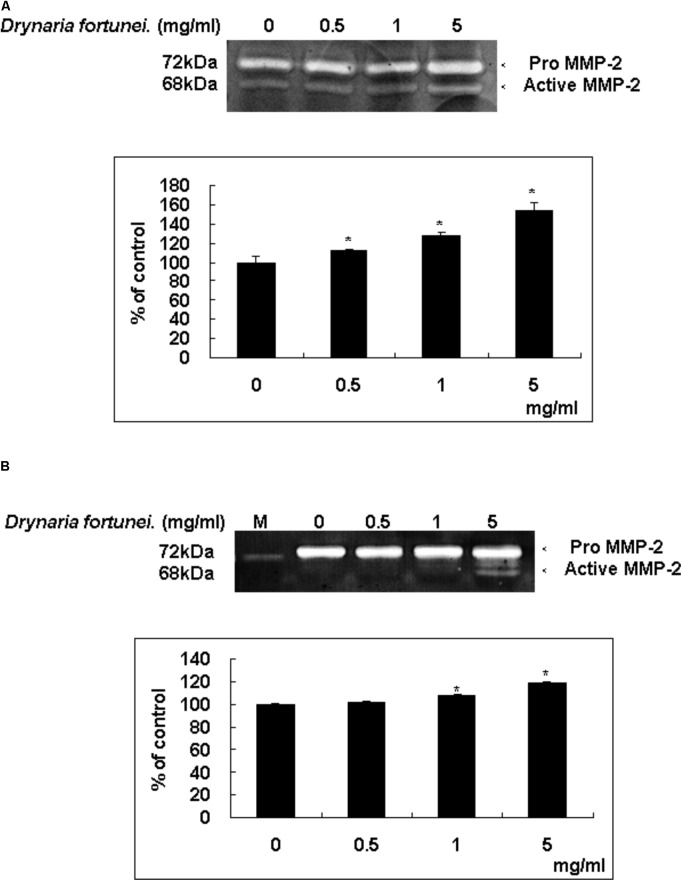
*D. fortunei* activated the MMP-2 activity both *in vivo* and *in vitro*. The effect of MMP-2 activity with the treatment of *D. fortunei* was determined by gelatin zymography. The conditioned media were collected from **(A)** CAM of 10-day-old chick embryos and **(B)** HUVEC cells with different concentrations of *D. fortunei* for 24 h. White bands on blue backgrounds indicated zones of digestion corresponding to the presence of MMP-2. The active MMP-2 with densitometry analysis was enhanced associated with the increasing concentrations of *D. fortunei* both *in vivo* and *in vitro*. Data were mean ± SD calculated from three individual experiments. An asterisk demonstrated the significant difference (^∗^*P* < 0.05) compared with control. M indicated medium only without serum.

Zymography is a technique used to study hydrolytic enzymes on the basis of substrate degradation. In this study, the expressions of MMPs of CAMs after treatment with *D. fortunei* were analyzed by gelatin zymography. The dominant gelatin-type MMP from CAMs was MMP-2. In the presence of the aqueous extract of *D. fortunei*, treatment with 5 mg/ml *D. fortunei* increased MMP-2 activity compared to the basal levels (**Figure 5A**). Conditioned media was collected from cultured HUVECs after treatment with different concentrations of *D. fortunei* for 24 h. The functional MMP-2 level increased proportionally as the dose of *D. fortunei* was enhanced, up to 5 mg/ml (**Figure 5B**). It indicated that treatment with *D. fortunei* on the CAMs and HUVECs promoted the expression of functional MMP-2 activity.

### *D. fortunei* Increased MMP-2, MMP-14 and Decreased TIMP-2, RECK Expressions Both in mRNAs and Proteins

**FIGURE 6:**
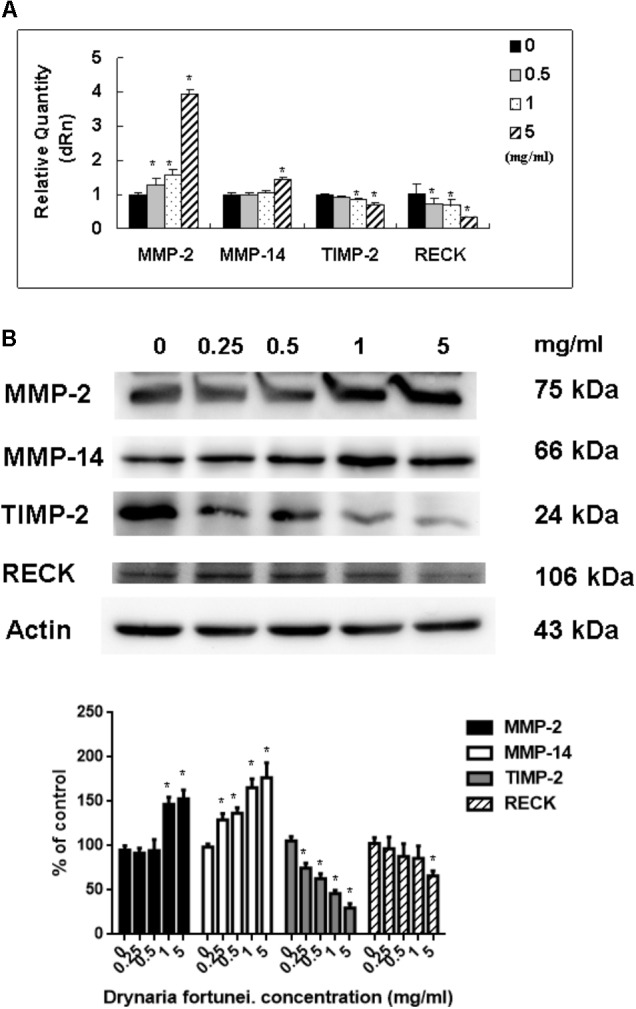
The measurement of mRNAs and proteins, including MMP-2, MMP-14, TIMP-2, and RECK by *D. fortunei.* **(A)** The relative quantity of mRNA, including MMP-2, MMP-14, TIMP-2, and RECK with different concentrations of *D. fortunei* as indicated. The mRNA levels of MMP-2 and MMP-14 were upregulated, while TIMP-2 and RECK were downregulated significantly by *D. fortunei*. HUVECs treated with or without *D. fortunei* for 24 h were processed for Western blot method to analyze the protein levels of MMP-2 and TIMP-2 in conditioned media without concentration as well as MMP-2, MMP-14, TIMP-2, and RECK in cytosol. **(B)** The protein levels of MMP-2 and MMP-14 in cytosol were increased. Conversely, the protein levels of TIMP-2 and RECK in cytosol were decreased. Data were mean ± SD calculated from three individual experiments. An asterisk demonstrated the significant difference (^∗^*P* < 0.05) compared with control.

As for the metalloproteinases family related to the process of angiogenesis, the MMP-2, MMP-14, TIMP-2, and RECK mRNA and protein levels were measured. Both the mRNA and protein levels of MMP-2 and MMP-14 were increased significantly after treatment with *D. fortunei* (**Figure 6A**). By contrast, the mRNA and protein levels of TIMP-2 and RECK decreased after treatment with *D. fortunei* (**Figure 6B**). The results indicated that *D. fortunei* upregulated MMP-2 and MMP-14, as well as downregulated TIMP-2 and RECK, both in transcription and translation levels.

### *D. fortunei* Affected VEGFs and VEGFRs Expressions

**FIGURE 7:**
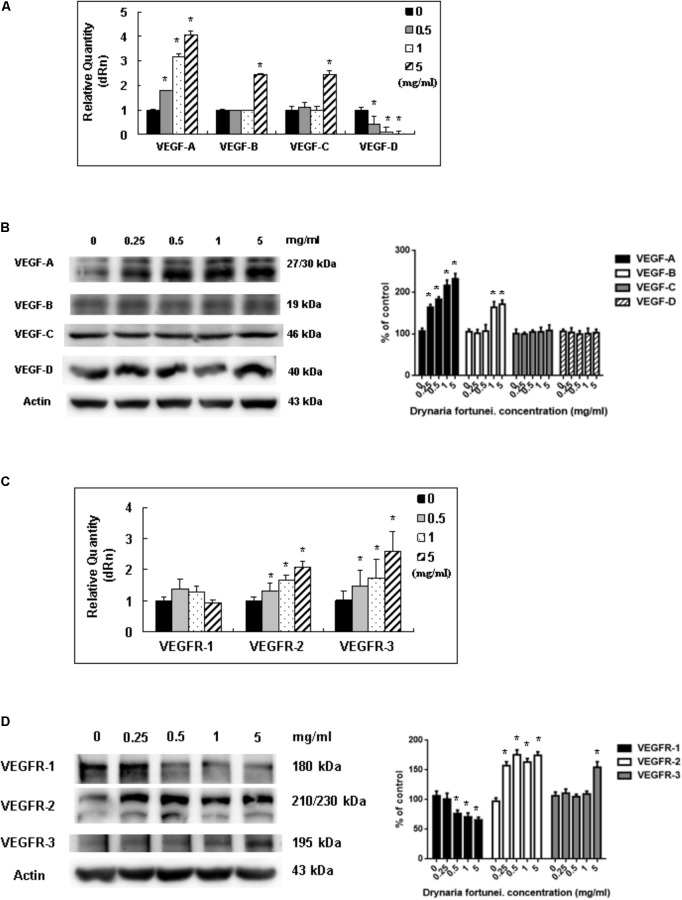
The measurement of mRNAs and proteins of VEGFs and VEGFRs by *D. fortunei.* The relative quantity of mRNA and protein expression of VEGFs and VEGFRs with different concentrations of *D. fortunei* as indicated. **(A)** The mRNAs of VEGF-A, -B, and -C were stimulated by *D. fortunei*; however, the mRNA of VEGF-D was suppressed. **(B)** As for the protein of VEGFs, the protein expressions of VEGF-A and VEGF-B were activated significantly by *D. fortunei* dose-dependently. However, the protein expression of VEGF-C and VEGF-D showed no significant change. **(C)** The mRNAs of VEGFR-2, and -3 were stimulated by *D. fortunei*; however, the mRNA of VEGFR-1 showed no change. **(D)** As for the protein of VEGFRs, the protein expressions of VEGFR-2 and VEGFR-3 were activated significantly by *D. fortunei* dose-dependently. However, the protein expression of VEGFR-1 was slightly decreased. Data were mean ± SD calculated from three individual experiments. An asterisk demonstrated the significant difference (^∗^*P* < 0.05) compared with control.

Vascular endothelial growth factors and VEGFRs are the most important ligand/receptors in the process of angiogenesis to promote wound healing. In this study, the mRNA and protein levels of VEGF-A increased significantly after treatment with *D. fortunei* (**Figures 7A,B**). The mRNA levels of VEGF-B and VEGF-C were also enhanced, but only when the concentration of *D. fortunei* was up to 5 mg/ml. On the contrary, the mRNA level of VEGF-D decreased (**Figure 7A**). The protein level of VEGF-B was also activated by *D. fortunei*; while, neither the protein levels of VEGF-C nor VEGF-D were activated (**Figure 7B**). Of note, the mRNA and protein expressions of VEGFR-2 and VEGFR-3 were enhanced after the treatment of *D. fortunei*, but no significant activation of VEGFR-1 mRNA or protein expressions were observed (**Figures 7C,D**). The original images of Western blots including MMP-2, MMP-14, TIMP-2, RECK, VEGF/VEGFRs and actin were shown in **Supplementary Figure S2**.

### MMP-2 and VEGF Inhibitors Suppressed Angiogenesis and Migration Induced by *D. fortunei*

**FIGURE 8:**
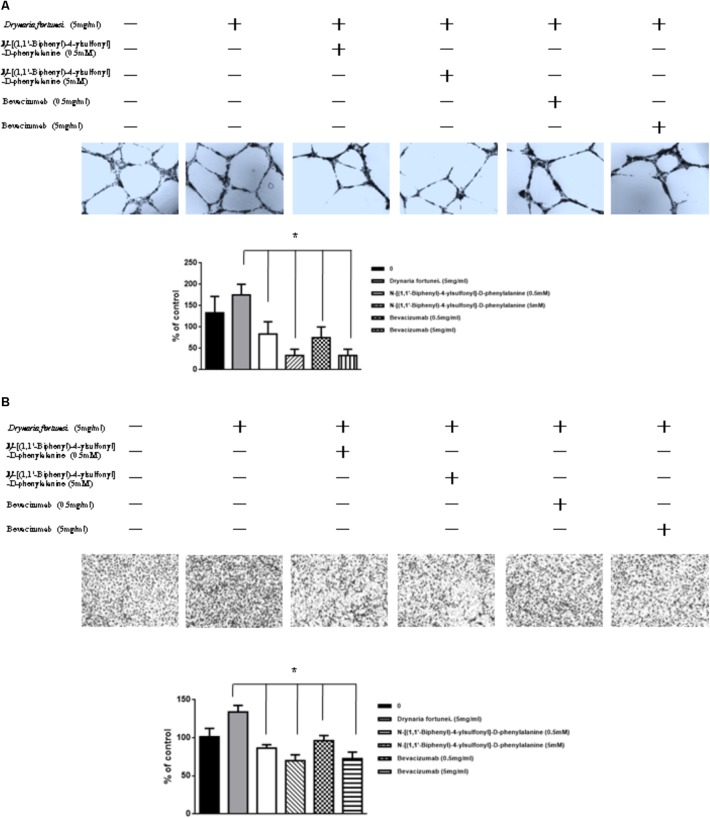
Inhibitors of MMP-2 and VEGF to suppress the angiogenic effects induced by *D. fortunei. N*-[(1,1’-biphenyl)-4-ylsulfonyl]-D-phenylalanine (MMP-2 inhibitor) and bevacizumab (VEGF inhibitor) blocked *D. fortunei*-induced transwell tube formation **(A)** and endothelial cell migration **(B)**. Data were mean ± SD calculated from three individual experiments. An asterisk demonstrated the significant difference (^∗^*P* < 0.05) compared with control.

**FIGURE 9:**
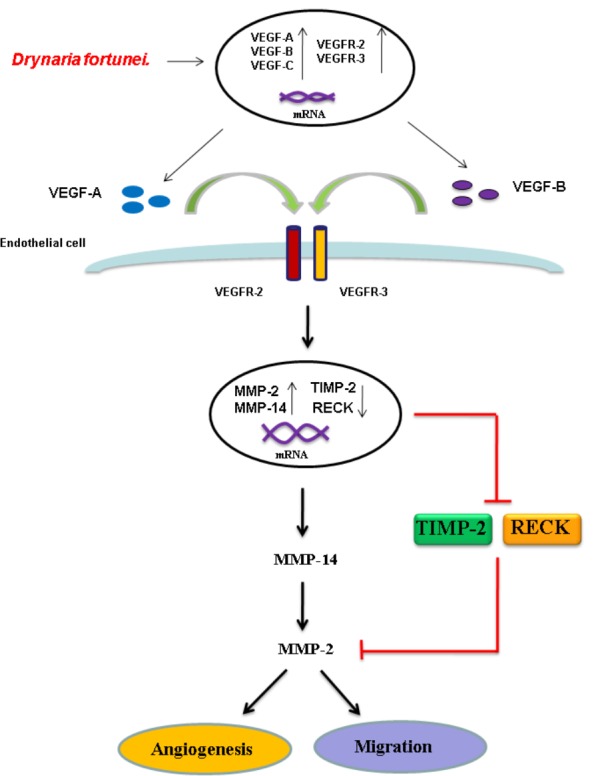
Schematic diagram of *D. fortunei-*induced angiogenesis through modulation of MMP-2/TIMP-2 balance and activation of VEGF ligand/receptors expressions.

To further assess the relevance of the results related to angiogenesis and migration, we examined the inhibitory effects of MMP and VEGF axis through the use of *N*-[(1,1′-biphenyl)-4-ylsulfonyl]-D-phenylalanine (MMP-2 inhibitor) and bevacizumab (VEGF inhibitor). We examined the transwell tube formation and migration induced by *D. fortunei* with treatment of both inhibitors. We found that *N*-[(1,1′-biphenyl)-4-ylsulfonyl]-D-phenylalanine and bevacizumab significantly inhibited angiogenesis and migration activities induced by *D. Fortunei* (**Figures 8A,B**). To illustrate our findings, we created a schematic diagram to show how *D. fortunei* effectively modulated MMP-2/TIMP-2 balance and augmented VEGF/VEGFRs interaction (**Figure 9**).

## Discussion

Phytochemical studies have demonstrated that flavonoids, phenylpropanoids, and triterpenes are major compounds present in *D. fortunei* (Wang et al., 2008; Qiao et al., 2014). However, cultivation methods and climate conditions may influence the quality of the rhizomes present in *D. fortunei*. A study by Liu et al. (2012) revealed the water extract of *D. fortunei* to be abundant in caffeic acid 4-*O*-β-D-glucopyranoside (**2**), 5,7-dihydroxychromone-7-*O*-rutinoside (**3**), neoeriocitrin (**4**) and naringin (**5**). We tentatively identified compounds **2**, **4**, and **5** according to their respective intensity compared to EIC; whereas compound **3** is **3**-**3** in **Figure 1B**. Our study also showed that the aqueous extract contained protocatechuic acid (**1**), at 0.07% (w/w), based on extract. Further study is imperative to delineate the specific compounds and to determine potential interactions of those compounds found in *D. fortunei*.

D. fortunei is one of the most commonly prescribed Chinese herbal medicines for wound healing. There are more than 300 constituent compounds which may be extracted from D. fortunei, including flavonoids, phenolcarboxylic acids, glycosides, and triterpenoids (Qiao et al., 2014; Han et al., 2015). In this study, no cytotoxic effects on HUVECs were identified, which is in accordance with the description found in Compendium of Materia Medica. Several studies have shown that D. fortunei could enhance the osteoblast activity and inhibit the osteoclast function associated with the promotion of protein phosphorylation in p38MAPK and ERK1/2 (Sun et al., 2004; Li et al., 2006; Wang et al., 2011; Zhu et al., 2012). However, while a thorough understanding of the angiogenic mechanisms and wound healing activities of D. fortunei is still lacking, this is the first study to effectively demonstrate that D. fortunei influences cell regrowth and migration to promote wound healing. Endothelial cell migration is essential in the wound healing process. Indeed, the major mechanisms involved in endothelial cell migration are: chemotaxis, the directional migration toward a gradient of soluble chemoattractants; and mechanotaxis, the directional migration generated by mechanical forces (Lamalice et al., 2007). Our results show that treatment of D. fortunei to the scratch wound area enhances cell filling of the cleared space of HUVECs; more specifically, the chemotaxis migration of HUVECs was augmented after D. fortunei treatment. This finding indicates that D. fortunei has the potential to accelerate wound closure, increase angiogenesis, favorably regulate ECM remodeling, and encourage regeneration of skin with normal architecture and function.

Several key ingredients in *D. fortunei* are critical in influencing bone development; specifically, the flavonoid naringin, which is a major compound found in *D. fortunei*. It has been reported to enhance osteoblast proliferation by augmenting the expression of BMP-2 and inhibiting osteoclast activity by reducing the expression of RANKL (Yin et al., 2015). Recently, Zhao et al. (2018) found that naringin enhanced EPC proliferation and tube formation mediated by the activation of the PI3K/Akt signaling pathway via the CXCL12/CXCR4 axis. Besides, naringin improved random skin flap survival and osteoporosis through promoting angiogenesis by regulating the VEGF/VEGFR signaling pathway and inhibiting inflammation by down-regulation of TNF-α and IL-6 (Cheng et al., 2017; Song N. et al., 2017). PA is another major ingredient present in *D. fortunei*, which has been shown in this study to promote angiogenesis and cell migration. In this study, we found that *D. fortunei* water extract containing the similar concentration of PA to exert the angiogenesis, migration, and wound healing process. The possible molecular mechanism affecting the angiogenic properties of PA is via a programmed PI3K/Akt/eNOS/VEGF signaling axis which promotes neovascularization and invasion (Kang et al., 2013; Song Y. et al., 2017). Moreover, PA had a strong inhibitory effect on fibroblast over-proliferation and keloid formation (Phan et al., 2003). As for the other ingredients, such as caffeic acid 4-*O*-β-D-glucopyranoside, 5,7-dihydroxychromone-7-*O*-rutinoside, and neoeriocitrin, there are as yet no reports related to the wound healing process or angiogenesis. Pectins and sugars are ubiquitous in aqueous extract in medicinal plants. Those hydrophilic constituents will be eluted out fast and RT is pretty short without UV absorption. We don’t expect that pectins and sugars will contribute the angiogenesis effect. However, it is important to note that further investigation into the interactions of ingredients present in *D. fortunei*.

Angiogenesis plays a critical role in the process of wound healing during which angiogenic capillary sprouts invade the wound clot and organize into a microvascular network throughout the granulation tissue (Tonnesen et al., 2000). The process of angiogenesis is mediated by dynamic interactions of endothelial cells with the ECM environment and angiogenic cytokines, such as MMPs, TIMP, and VEGFs (Maxson et al., 2012; Lancerotto and Orgill, 2014). Essentially, the endothelial cells are activated, degrade the basement membrane, migrate into the injured ECM, remodel morphology into tubular structures, and form the first primitive vascular plexus in the wound tissue. For study purposes, the *ex vivo* CAM assay and *in vivo* DIVAA mimic the physiological cellular environment, allowing for investigation into angiogenesis. The CAM is an extra embryonic membrane which is composed of multilayer epithelium, ectoderm, mesoderm, and endoderm (Valdes et al., 2002). In the present study, increased vessel density was associated with increased concentrations of *D. fortunei*, in a dose-dependent manner. Additionally, *D. fortunei* promoted the tube formation of HUVECs via matrigel ECM gels. Our results demonstrate that *D. fortunei* effectively enhances angiogenesis, both *in vitro* and *in vivo*.

As for endothelial cell morphogenesis, endothelial tube formation within the matrigel ECM gel matrix has shown the *in vitro* angiogenic effects of *D. fortunei*. Matrix degradation, a critical step of angiogenesis, is the decline of the laminin-rich basement membrane and proteolysis of the collagen-rich ECM (Goodwin, 2007). Gelatin zymography assays in this study indicated that *D. fortunei* enhanced MMP-2 activity both *in vivo* and *in vitro*. MMP-2 is the most important of the MMPs in degrading the ECM during the course of endothelial cell migration for angiogenesis. MMP-2 is secreted as inactive zymogen (pro MMP-2; 72 kDa), and subsequently activated (active MMP-2; 68 kDa) (Lai et al., 2012). The expression and activation of MMP-2 are dose-dependently enhanced after treatment with *D. fortunei*. In addition, the expression of MMP-14, a main transmembrane MMP for angiogenesis, increased significantly under higher concentrations with *D. fortunei* treatment. Moreover, the expression of TIMP-2 and RECK, the main MMP inhibitors, demonstrated statistically significant decreases. MMP-2 is secreted as proMMP-2 and is later activated in association with MMP-14, TIMP-2, and RECK mechanisms to enhance angiogenesis, and thus induce wound healing. Fundamentally, the angiogenic mechanisms of *D. fortunei* are considered to involve the modulation of gene transcription and protein translation levels between MMP-2/TIMP-2 balances (**Figure 9**).

Vascular endothelial growth factor is a powerful angiogenic stimulatory factor critical to the cell migration process (Chodorowska et al., 2004). VEGF-A and VEGF-B are the key inducers of developmental angiogenesis via activation of VEGFR-1 and VEGFR-2. VEGF-C and VEGF-D have been identified as lymphatic endothelial factors, which act via VEGFR-3. As reported, the biology of the different VEGFR-2 and VEGFR-3 ligands overlaps quite extensively and both receptor types contribute to angiogenesis as well as lymphangiogenesis (Bahram and Claesson-Welsh, 2010). This study confirms the elevated expressions of VEGF-A, -B and VEGFR-2, -3 are associated with treatment of *D. fortunei*. It has been noted that *D. fortunei* could promote angiogenesis and vascular permeability by the VEGF/VEGFR system (Shibuya, 2013). Furthermore, we found that *D. fortunei* treatment is associated with elevated expressions of VEGF-C and VEGFR-3, combined with suppression of VEGF-D. The question of whether lymphangiogenesis is also affected by *D. fortunei* treatment requires further investigation. Besides, whether the concentration of water extract of *D. fortunei* or PA is available in physiological conditions is still ambiguous. It still needs further investigation in our future study.

## Conclusion

This is the first study to demonstrate that *D. fortunei* plays an essential role in angiogenesis, effectively promoting the cell migration process both *in vivo* and *in vitro*. The activation of cell migration and angiogenesis by *D. fortunei* is associated with the enhancement of MMP-2 activity in vascular endothelial cells; specifically through the modulation of MMP-2/TIMP-2 balance and augmentation of VEGF ligand/receptors interaction. We conclude that *D. fortunei* is highly effective in stimulating angiogenesis, and therefore offers promise as a therapeutic medicine for clinical application.

## Author Contributions

S-TH wrote the paper and conceived and conducted the entire study. C-CC wrote the paper and amended the references. J-HP edited the whole article and offered technique support. S-CC analyzed the fingerprint and confirmed the structures of extract. M-CK offered technique assistance and reviewed the text. H-SH and H-LY performed the experiments and analyzed the data. All authors have read and approved the manuscript.

## Conflict of Interest Statement

The authors declare that the research was conducted in the absence of any commercial or financial relationships that could be construed as a potential conflict of interest.

## Supplementary Material

FIGURE S1 *D. fortunei* without cytotoxic effect of HUVECs. **(A)** The cell viability was determined by standard MTT assay with different concentrations of *D. fortunei* as indicated for 24 h. **(B)** The cytotoxic effect of *D. fortunei* was evaluated by the LDH assay using the conditioned medium collected from HUVECs treated with different concentrations of *D. fortunei* as indicated for 24 h. **(C)** The cell viability was measured by MTT assay with different concentrations of PA as indicated for 24 h. No cytotoxicity of either *D. fortunei* or PA was detected under the experimental conditions. Data were mean ± SEM calculated from three individual experiments.

FIGURE S2 The original images of Western blots including MMP-2, MMP-14, TIMP-2, RECK, VEGF/VEGFRs, and actin.

FIGURE S3 The UV-Vis spectra for **3**-**1**, **3**-**2**, **3**-**3**, and **3**-**4**.

FIGURE S4 The MS, MS/MS for **1**-**5** and base peak chromatography (BPC) for water extract of *D. fortunei*.

TABLE S1 Primer sequences.

## Abbreviations

CAMs: chick chorioallantoic membranes
*D. fortunei*: *Drynaria fortunei*
DIVVA: directed *in vivo* angiogenesis assay
ECM: extracellular matrix
ESI: electrospray ionization
GMP: good manufacturing practice
HPF: high-power microscopic field
HUVEC: human umbilical vascular endothelial cell
LDH: lactate dehydrogenase
MMPs: matrix metalloproteinases
OD: optical density
PGF: placenta growth factor
PMA: phorbol-12-myristate-13-acetate
RECK: reversion-inducing cysteine-rich protein with kazal motifs
TCM: traditional Chinese medicine
TIMP: tissue inhibitor of metalloproteinase
VEGF: vascular endothelial growth factor
