# Supplementary material for: Drynaria fortunei Promoted Angiogenesis Associated With Modified MMP-2/TIMP-2 Balance and Activation of VEGF Ligand/Receptors Expression
Source: Front Pharmacol. 2018 Sep 21;9:979. doi: 10.3389/fphar.2018.00979 (PMC6160574; doi:10.3389/fphar.2018.00979)

Supplementary Fig. 1

(A)

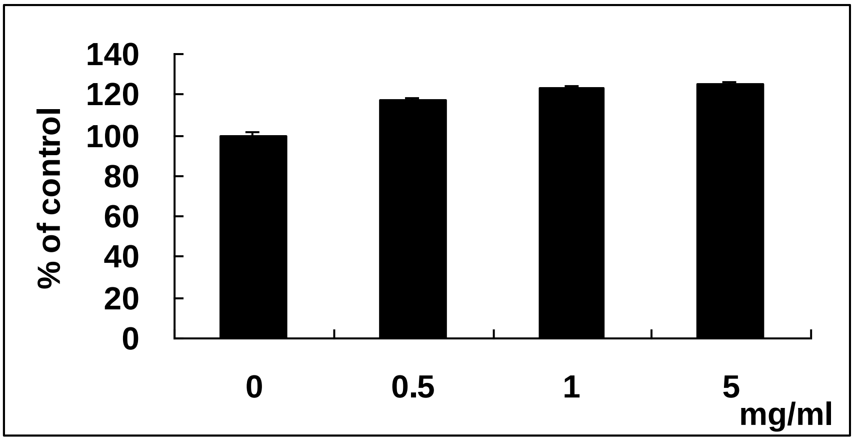


(B)

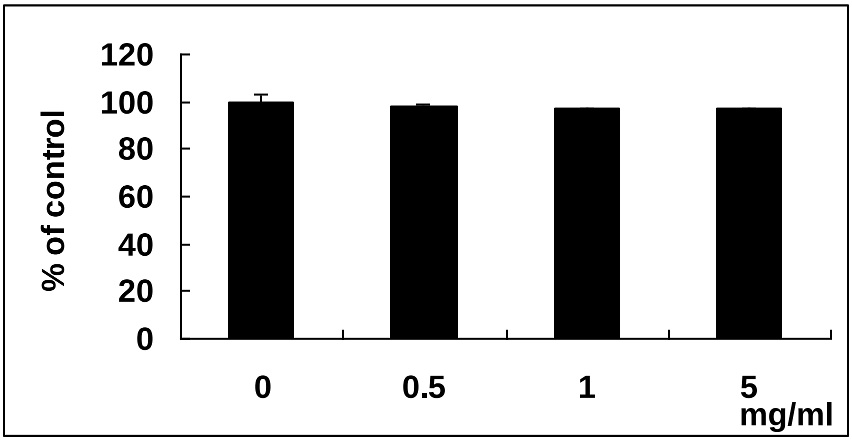


(C)

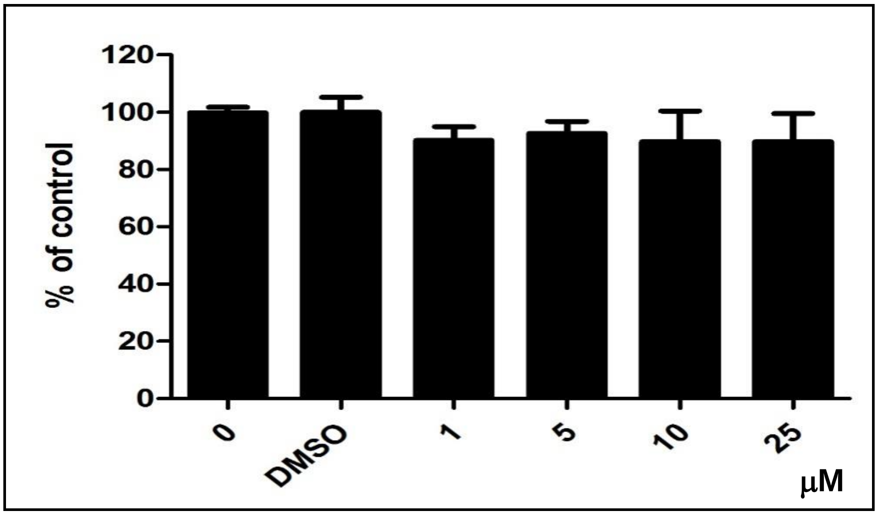

# Supplementary Fig. 2

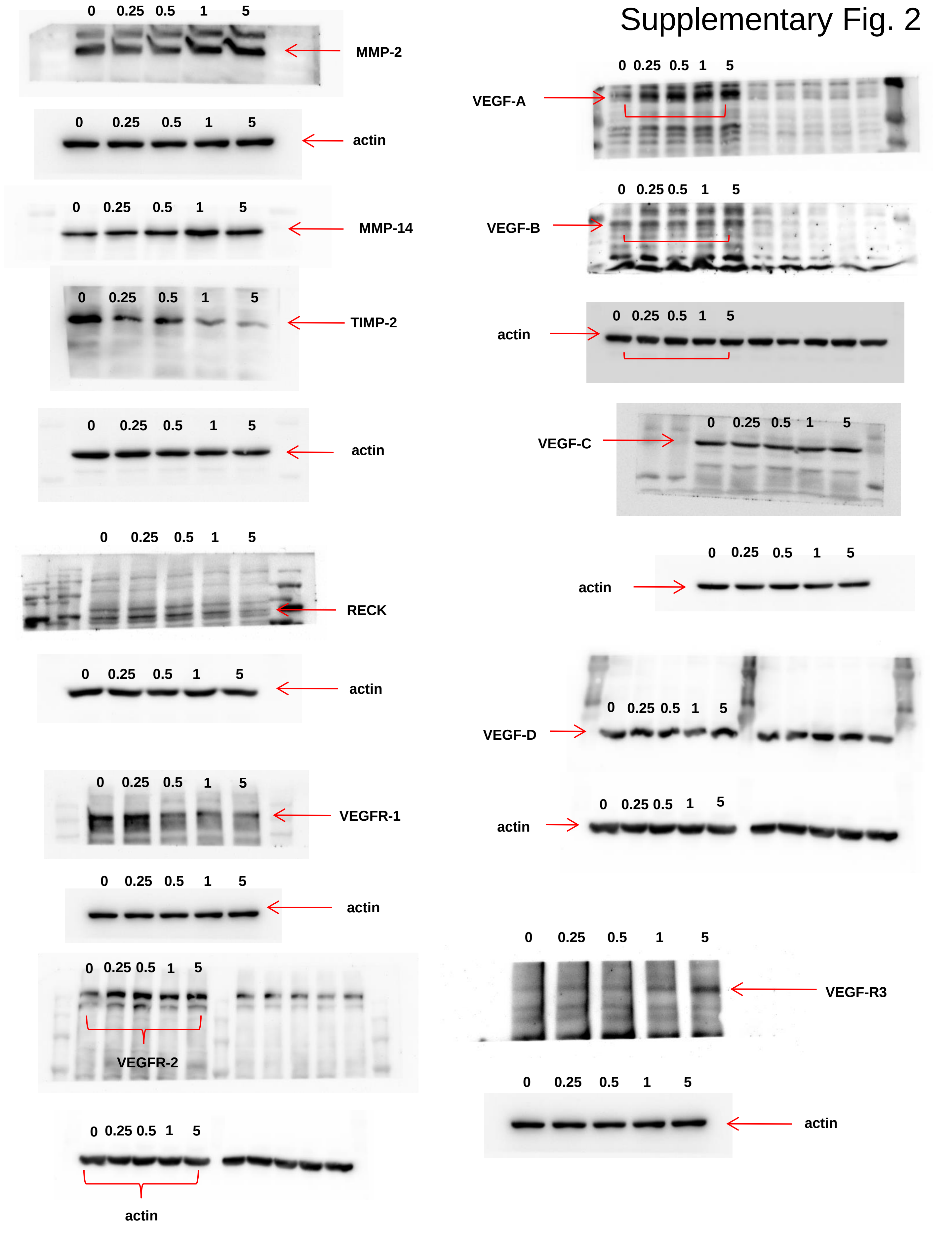

# Supplementary Fig. 3

3-1

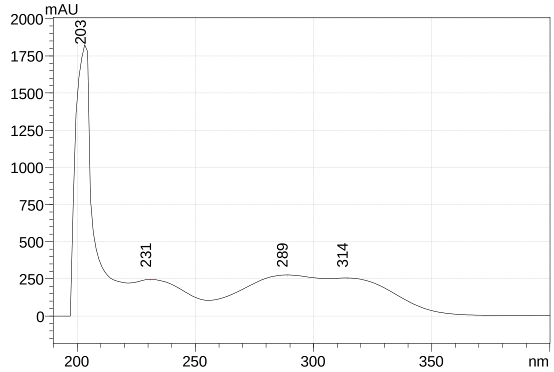


3-2

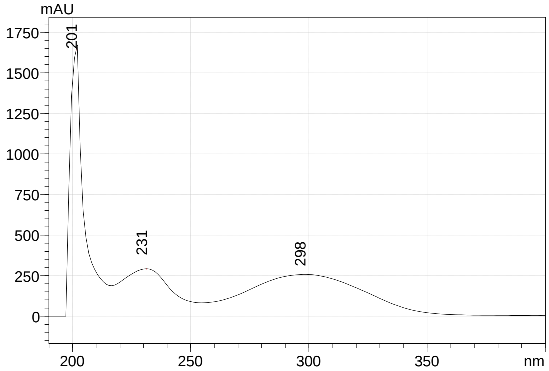


3-3

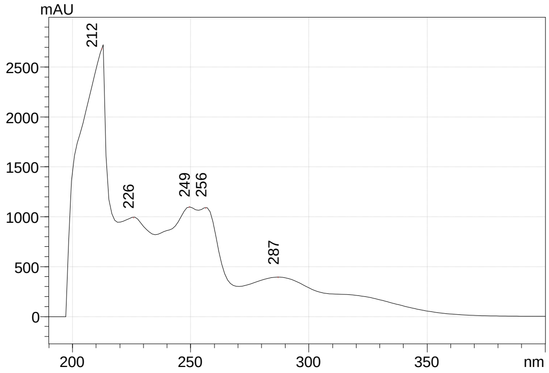


3-4

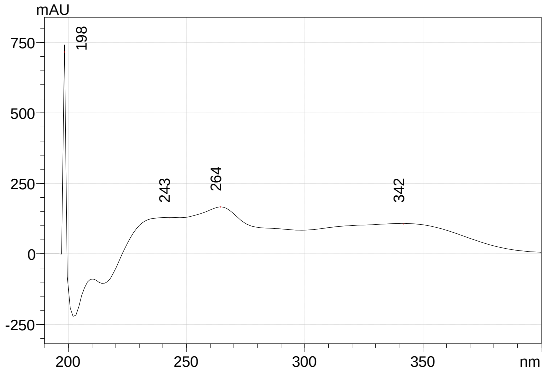

# Supplementary Fig. 4

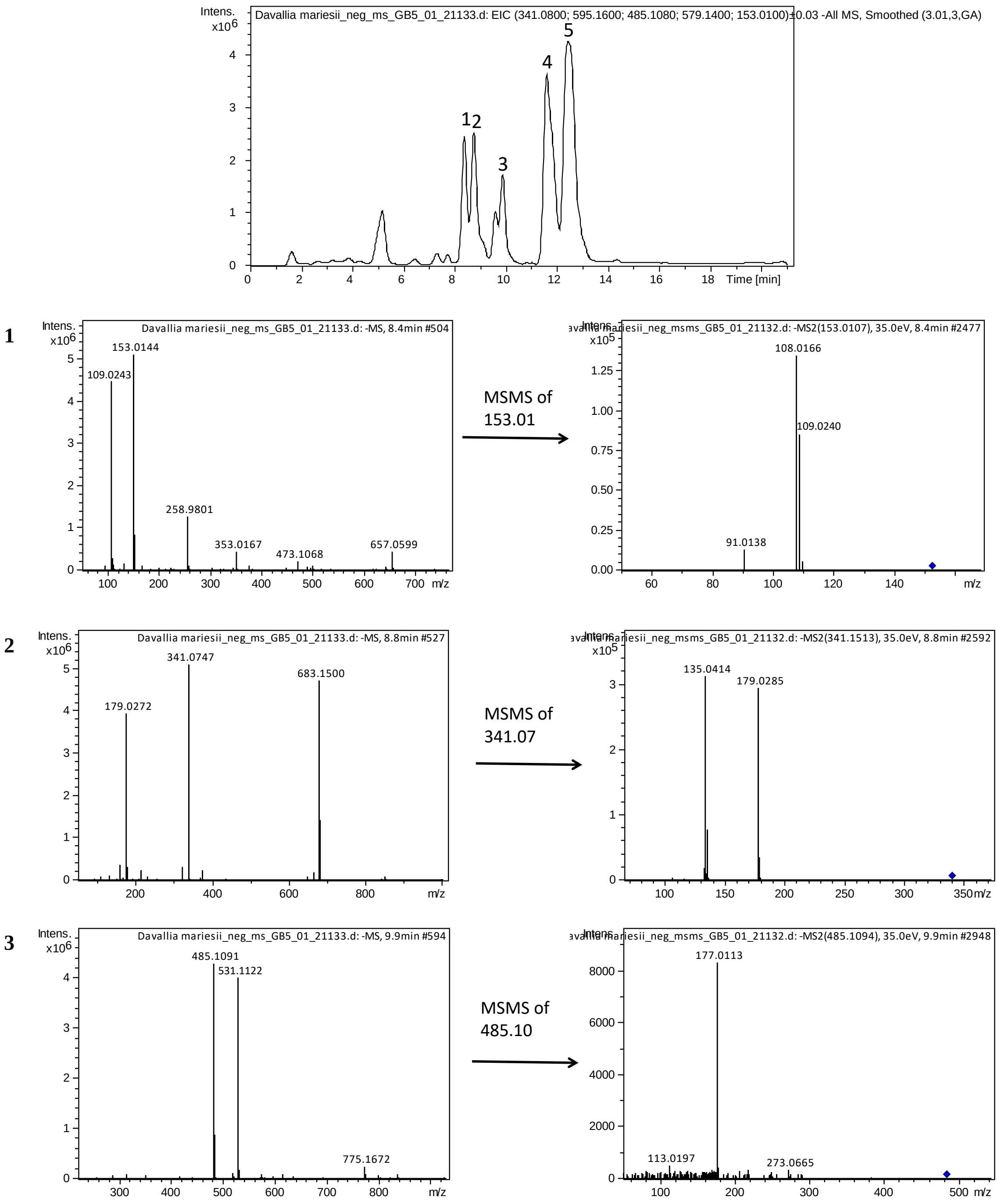

# Supplementary Fig. 4, continued.

4

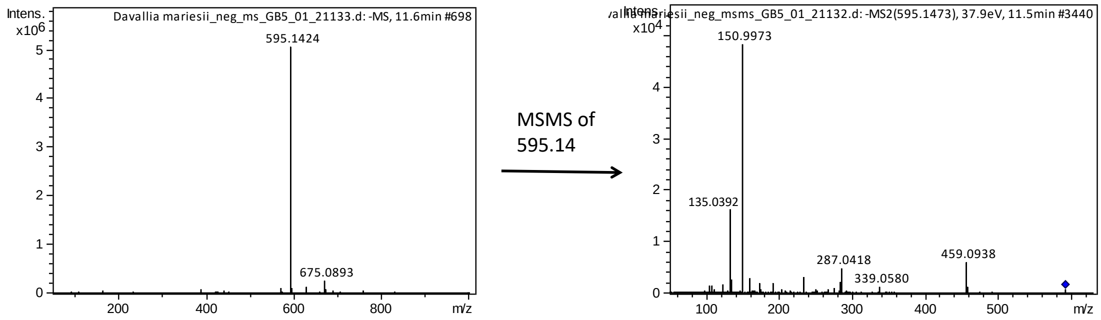


5

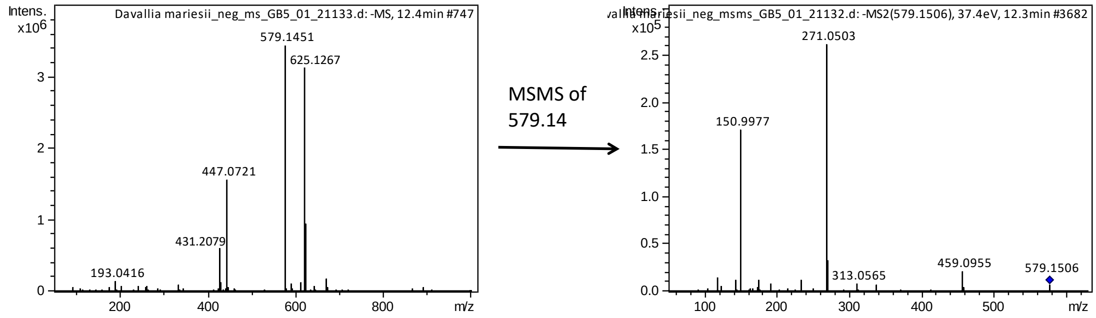


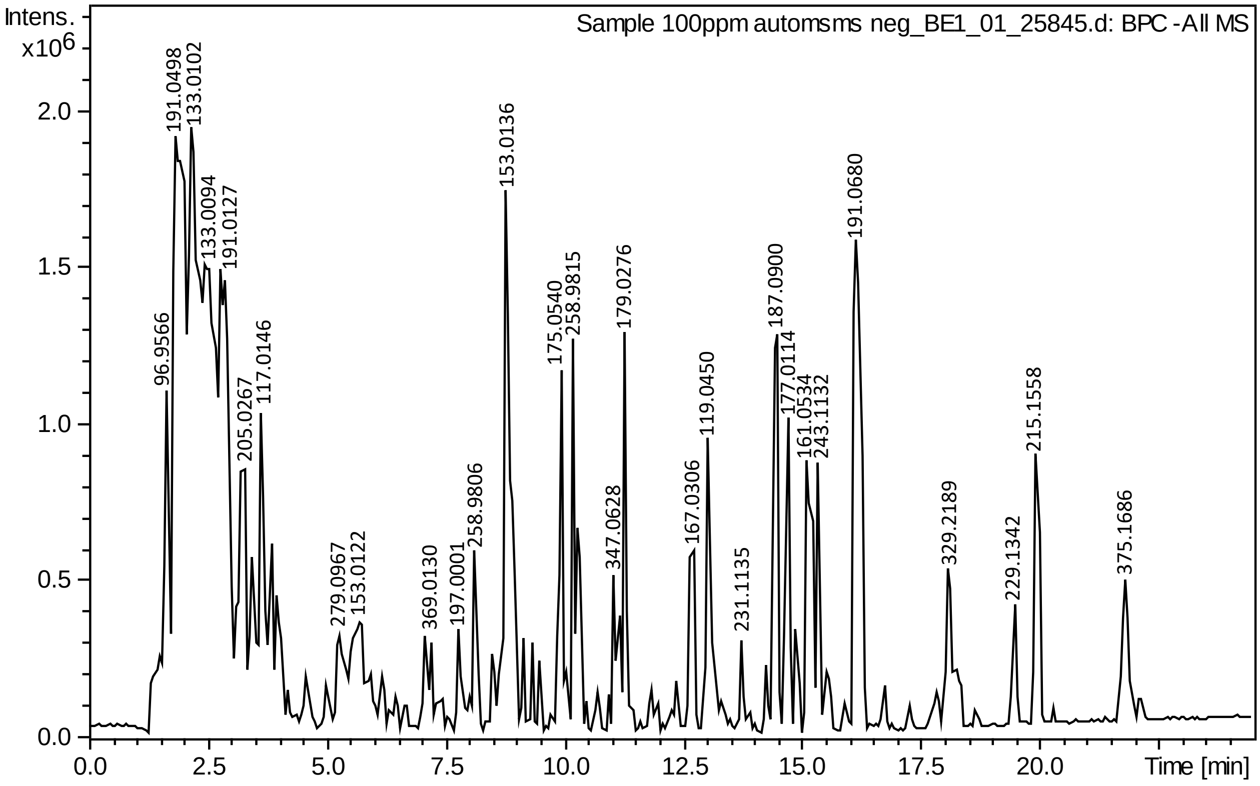

Supplement: FIGURE S1 — D. fortunei without cytotoxic effect of HUVECs. (A) The cell viability was determined by standard MTT assay with different concentrations of D. fortunei as indicated for 24 h. (B) The cytotoxic effect of D. fortunei was evaluated by the LDH assay using the conditioned medium collected from HUVECs treated with different concentrations of D. fortunei as indicated for 24 h. (C) The cell viability was measured by MTT assay with different concentrations of PA as indicated for 24 h. No cytotoxicity of either D. fortunei or PA was detected under the experimental conditions. Data were mean ± SEM calculated from three individual experiments. [file Data_Sheet_1.PDF]
