# Supplementary material for: Drynaria fortunei Promoted Angiogenesis Associated With Modified MMP-2/TIMP-2 Balance and Activation of VEGF Ligand/Receptors Expression
Source: Front Pharmacol. 2018 Sep 21;9:979. doi: 10.3389/fphar.2018.00979 (PMC6160574; doi:10.3389/fphar.2018.00979)
Supplement: TABLE S1 — Primer sequences. [file Table_1.DOC]

| **Supplementary Table 1 | Primer sequences** | |
| --- | --- |
| GAPDH | Sense:5’-GACCTGACCTGCCGTCTA-3’ |
|  | Antisense:5’-AGGAGTGGGTGTCGCTGT-3’ |
| MMP-2 | Sense: 5’-GTTTCCATTCCGCTTCCAGG-3’ |
|  | Antisense:5’-TGCCCTTGATGTCATCCTGG-3’ |
| MMP-14 | Sense: 5’-GCCTTGGACTGTCAGGAATG-3’ |
|  | Antisense:5’-AGGGGTCACTGGAATGCTC-3’ |
| TIMP-2 | Sense: 5’-GAAGAGCCTGAACCACAGGT-3’ |
|  | Antisense:5’-CGGGGAGGAGATGTAGCAC-3’ |
| RECK | Sense: 5’-CAAGTGTCCTTCGCTCTTGG-3’ |
|  | Antisense:5’-CACATAATGGGCAACAAGCA-3’ |
| VEGF-A | Sense: 5’-ATTATGCGGATCAAACCT-3’ |
|  | Antisense: 5’-TTCTTGTCTTGCTCTATCTT-3’ |
| VEGF-B | Sense: 5’-AGATGTCCCTGGAAGAACACA-3’ |
|  | Antisense: 5’-GGGCTGTCTGGCTTCACA-3’ |
| VEGF-C | Sense: 5’-GTGTCCAGTGTAGATGAA-3’ |
|  | Antisense: 5’-CCTGTTCTCTGTTATGTTG-3’ |
| VEGF-D | Sense: 5’-TACCAACACATTCTTCAA-3’ |
|  | Antisense: 5’-CATACAGATAAGGCTCTC-3’ |
| VEGFR-1 | Sense: 5’-CTCCATCACTCTTAATCTTAC-3’ |
|  | Antisense: 5’-TATACATTCCTGGCTCTG-3’ |
| VEGFR-2 | Sense: 5’-CCAAGAAGAACAGCACAT-3’ |
|  | Antisense: 5’-TTCCACCAGAGATTCCAT-3’ |
| VEGFR-3 | Sense: 5’-GAGGGAAAGAATAAGACT-3’ |
|  | Antisense: 5’-GGTCACATAGAAGTAGAT-3’ |
